# Sex Differences in Right Ventricular Dysfunction: Insights From the Bench to Bedside

**DOI:** 10.3389/fphys.2020.623129

**Published:** 2021-01-18

**Authors:** Jennifer Keen, Sasha Z. Prisco, Kurt W. Prins

**Affiliations:** ^1^Pulmonary and Critical Care, Department of Medicine, University of Minnesota, Minneapolis, MN, United States; ^2^Cardiovascular Division, Department of Medicine, Lillehei Heart Institute, University of Minnesota, Minneapolis, MN, United States

**Keywords:** right ventricle, sex differences, pulmonary hypertension, arrhythmogenic right ventricular cardiomyopathy/dysplasia, estrogen, testosterone, dehydroepiandrosterone

## Abstract

There are inherent distinctions in right ventricular (RV) performance based on sex as females have better RV function than males. These differences are magnified and have very important prognostic implications in two RV-centric diseases, pulmonary hypertension (PH), and arrhythmogenic right ventricular cardiomyopathy/dysplasia (ARVC/D). In both PH and ARVC/D, RV dysfunction results in poor patient outcomes. However, there are no currently approved therapies specifically targeting the failing RV, an important unmet need for these two life-threatening disorders. In this review, we highlight human data demonstrating divergent RV phenotypes in healthy, PH, and ARVC/D patients based on sex. Furthermore, we discuss the links between estrogen (the female predominant sex hormone), testosterone (the male predominant sex hormone), and dehydroepiandrosterone (a precursor hormone for multiple sex hormones in males and females) and RV function in both disorders. To provide potential mechanistic insights into sex differences in RV function, we review data that investigate how sex hormones combat or contribute to pathophysiological changes in the RV. Finally, we highlight the ongoing clinical trials in pulmonary arterial hypertension targeting estrogen and dehydroepiandrosterone signaling. Hopefully, a greater understanding of the factors that promote superior RV function in females will lead to novel therapeutic approaches to combat RV dysfunction in PH and ARVC/D.

## Introduction

The right ventricle (RV) is a thin-walled, low pressure chamber that is derived from the pharyngeal mesoderm of the anterior heart field (Zaffran et al., 2004). In adults, the RV functions to transmit blood from the venous system to the pulmonary vasculature to permit gas exchange (Weber et al., 1983). Proper RV function is crucial for optimal cardiovascular health, and RV dysfunction results in poor outcomes in two RV-centric diseases, pulmonary hypertension (PH) (Riedel et al., 1982; Thenappan et al., 2010; Mohammed et al., 2014; Prins et al., 2018) and arrhythmogenic right ventricular cardiomyopathy/dysplasia (ARVC/D) (Cadrin-Tourigny et al., 2019). Unfortunately, research on the mechanisms underlying RV dysfunction has lagged behind our understanding of left ventricular (LV) dysfunction as demonstrated by the fact that there are currently multiple therapies with survival benefits for LV failure (Yancy et al., 2017; Lam et al., 2019), but there are no approved drugs for RV failure. Moreover, medications with proven survival benefits for LV failure, such as beta-blockers and renin-angiotensin-aldosterone inhibitors, have no or limited success in RV failure (Leier et al., 1983; van Campen et al., 2016). Furthermore, aldosterone antagonists do not seem to directly enhance RV function in preclinical studies (Boehm et al., 2018). The inability to translate therapies for LV failure to the dysfunctional RV may be due to developmental, anatomical, and functional differences between the RV and LV, which are reviewed elsewhere (Prisco et al., 2020). Consequently, further research is needed to define novel pathways that can be exploited to combat RV dysfunction to improve outcomes in PH and ARVC/D patients.

An estimated 1% of the global population and up to 10% of individuals >65 years old (Hoeper et al., 2016) have PH. PH is defined by a mean pulmonary artery pressure >20 mmHg (Simonneau et al., 2019). Clinically, PH is classified into five groups: World Health Organization (WHO) Group (1) pulmonary arterial hypertension (PAH); (2) PH due to left heart disease; (3) PH caused by lung diseases and/or hypoxia; (4) chronic thromboembolic PH (CTEPH) and other pulmonary artery obstructions; and (5) PH due to unclear and/or multifactorial mechanisms (Simonneau et al., 2019). Regardless of etiology, PH increases RV afterload and ultimately leads to RV failure and death. RV dysfunction is the strongest predictor of mortality in PAH (Benza et al., 2010; Humbert et al., 2010b; Thenappan et al., 2010; Steiner et al., 2015). Moreover, presence of RV failure is predictive of adverse outcomes in Group 2, 3, and 4 PH (Riedel et al., 1982; Mohammed et al., 2014; Prins et al., 2018). Despite the clear prognostic implications of RV dysfunction in multiple forms of PH, there are currently no approved therapies that directly target the RV.

ARVC/D is a rare but lethal hereditary disease that primarily affects the RV (Dalal et al., 2005). ARVC/D is characterized by fibro-fatty replacement of RV myocardium leading to progressive RV dysfunction, ventricular arrhythmias, and an increased risk of sudden cardiac death (Lin et al., 2017). The estimated prevalence of ARVC/D is 1:5,000 (Gemayel et al., 2001) and ARVC/D accounts for up to 20% of cases of sudden cardiac death in young athletes (Corrado et al., 1998; Tabib et al., 2003). Mutations in genes encoding the desmosomal proteins, plakophilin-2 (*PKP2*), desmoglein-2 (*DSG2*), desmocollin-2 (*DSC2*), desmoplakin (*DSP*), and junction plakoglobin (*JUP*), are the most common causes of hereditary ARVC/D (Ohno, 2016; Austin et al., 2019).

**Figure 1:**
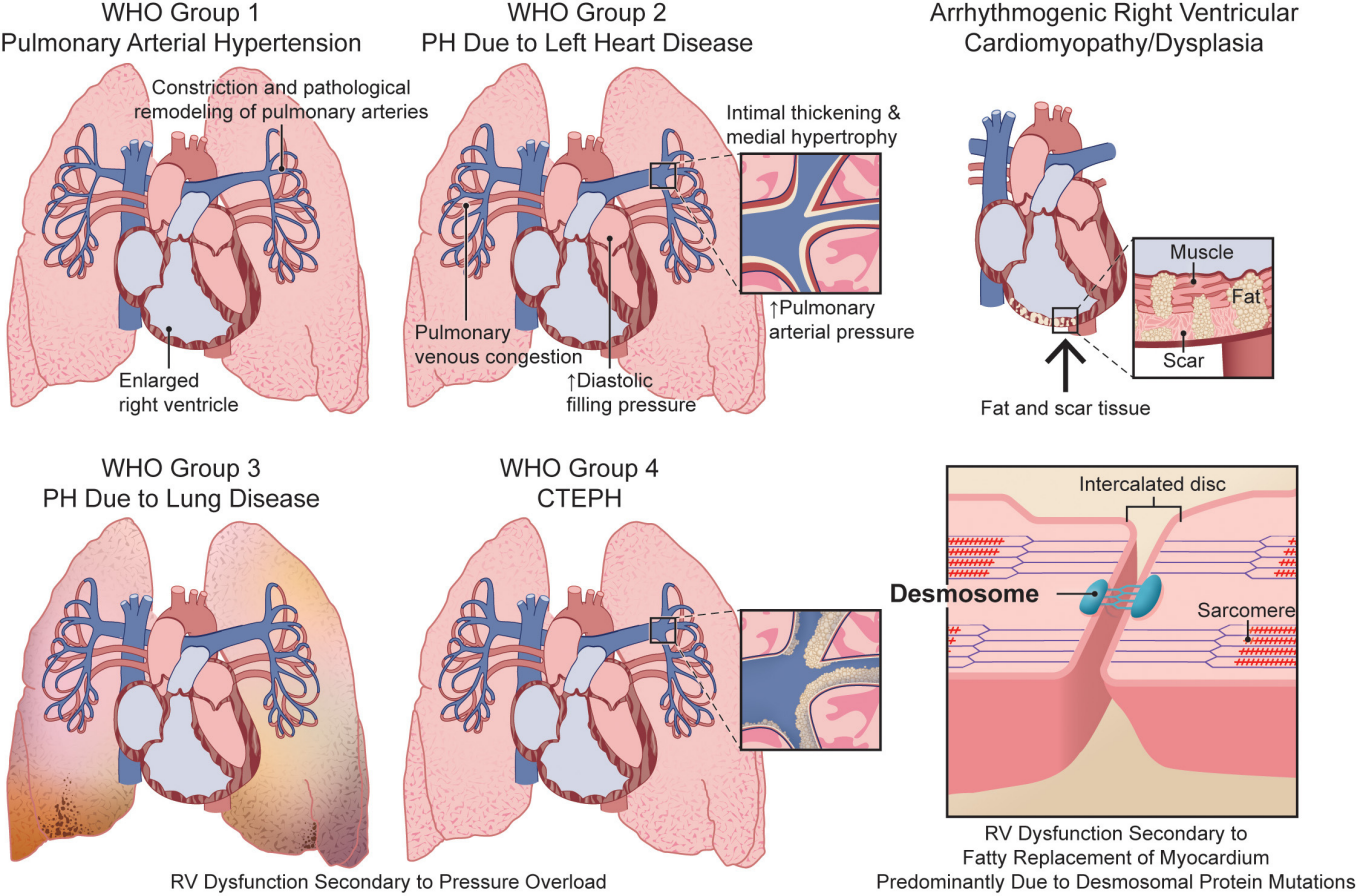
Comparison of the etiologies of right ventricular dysfunction in pulmonary hypertension and arrhythmogenic right ventricular cardiomyopathy/dysplasia. CTEPH, chronic thromboembolic pulmonary hypertension; PH, pulmonary hypertension; RV, right ventricular; WHO, World Health Organization.

Although PH and ARVC have distinct triggers that ultimately manifest as RV dysfunction (Figure 1), both disease entities exhibit important sex differences in RV phenotypes and survival. In this review, we highlight the clinical data demonstrating divergent RV phenotypes in healthy, PH, and ARVC/D patients based on sex. Furthermore, we review the studies showing sex hormones modulate pathophysiological mechanisms underlying RV dysfunction in PH and ARVC/D. Finally, we discuss the ongoing clinical trials in PAH attempting to modulate sex hormone physiology. Advancing our understanding of sex differences and mechanisms by which sex hormones modulate RV function may lead to new therapeutic approaches in PH and ARVC/D.

## Sex Differences in RV Function in Healthy Individuals

Distinct RV phenotypes based on sex are well-documented, and studies consistently demonstrate females have better RV systolic function than males. In the Multi-Ethnic Study of Atherosclerosis (MESA), a prospective study of men and women age 45–84 years old who are free of clinical cardiovascular disease, men have higher RV mass (by ~8%, *p* < 0.001) and volumes (by 7%, *p* < 0.001) and lower RV ejection fraction (absolute decrease of 4%, *p* < 0.001) as determined by cardiac magnetic resonance imaging (CMR). These differences are consistent in all ethnic groups studied (Caucasians, African Americans, Hispanics, and Chinese Americans) (Tandri et al., 2006; Kawut et al., 2011). Importantly, superior RV function in females persists even after multivariate adjustment for LV function and body size (Tandri et al., 2006; Kawut et al., 2011). Interestingly, the dissimilarities in RV size and function in healthy individuals parallel the trends observed in the LV with men having greater LV mass and volumes (Salton et al., 2002) and lower LV ejection fraction compared to women (Chung et al., 2006).

## The Sex Paradox in PAH (Group 1 PAH)

PAH is a progressive pulmonary arterial vasculopathy that ultimately leads to RV failure and death (Prins and Thenappan, 2016). In PAH, there is a sex paradox as the prevalence of PAH is greater in females than males (~3–4:1) (Benza et al., 2010; Hoeper et al., 2013), but females have better survival compared to males (hazard ratio [HR] 0.375, 95% confidence interval [CI] 0.212–0.662; *p* < 0.001) (Benza et al., 2010; Humbert et al., 2010a; Hoeper et al., 2013). The survival difference is predominately explained by enhanced RV function in females, which accounts for 39% of the difference in transplant-free survival between men and women (57 vs. 85% transplant-free survival at 5 years; *p* = 0.002) (Jacobs et al., 2014). In PAH, male sex is associated with lower RV ejection fraction as assessed by CMR (40 ± 17 vs. 48 ± 18%; *p* = 0.045) (Swift et al., 2015) and equilibrium radionuclide angiography (~25 vs. ~31%; *p* = 0.02), even after adjusting for pulmonary vascular resistance and LV ejection fraction (Kawut et al., 2009). A recent and important study documented that women with PAH have improved RV contractility (Ees) (0.7 ± 0.30 vs. 0.46 ± 0.23 mmHg/mL; *p* = 0.01) and RV-pulmonary artery coupling (Ees/effective arterial elastance [Ea]) (0.85 [0.56–1.41] vs. 0.52 [0.29–0.74]; *p* = 0.01; data presented as median [interquartile range (IQR)]) despite equivalent afterload (Ea) (0.80 [0.56–0.96] vs. 0.72 [0.46–1.10] mmHg/mL; *p* = 0.97; data presented as median [IQR]) (Tello et al., 2020). Finally, females exhibit a more favorable RV response to medical therapy as men have a disproportionate deterioration of RV function (RV ejection fraction −8.1%, 95% CI: −14 to −2; *p* < 0.01) despite similar reductions in pulmonary vascular resistance (−79 [−523 to +10] in men vs. −165 [−436 to +92] dyn × s × cm^5^ in women; *p* = 0.63; data shown as median [IQR]) (Jacobs et al., 2014). These studies suggest that sex hormones directly modulate RV function in PAH.

## Sex Differences in RV Function in Group 2 PH

Similarly, divergent RV phenotypes are observed in PH secondary to left heart disease (WHO Group 2), the most common cause of PH (Strange et al., 2012). In patients with heart failure with reduced ejection fraction, male sex is associated with more severe RV dilation (odds ratio [OR] 2.95, 95% CI: 1.09–8.03; *p* = 0.034) (Bourantas et al., 2011) and RV dysfunction (OR: 1.9, 95% CI: 1.1–3.5; *p* = 0.03) despite similar pulmonary systolic pressures, mean pulmonary pressures, pulmonary wedge pressures, and LV function (Martínez-Sellés et al., 2006). Furthermore, in patients with heart failure with preserved ejection fraction (HFpEF), male sex is an independent predictor of RV dysfunction and mortality (Melenovsky et al., 2014; Duca et al., 2018). Moreover, male HFpEF patients have lower RV fractional area change (RVFAC) at every mean pulmonary arterial pressure when compared to females (*p* < 0.0001) (Melenovsky et al., 2014).

## Females Have Superior RV Function in Group 3 PH

Patients with the second most common cause of PH, PH due to chronic lung disease (WHO Group 3), have the lowest survival rates of all PH groups (Strange et al., 2012; Wijeratne et al., 2018). RV dysfunction may contribute to this high mortality as we recently showed Group 3 PH patients have reduced RVFAC compared to Group 1 patients (28 ± 10 vs. 33 ± 11%; *p* = 0.006) despite having lower mean pulmonary arterial pressure (40 ± 10 vs. 47 ± 14 mmHg; *p* < 0.001) and pulmonary vascular resistance (6.9 ± 3.4 vs. 10.3 ± 5.6 Wood units; *p* < 0.001) (Prins et al., 2018). One possible explanation for this observation is that there is a higher proportion of males with Group 3 PH (males: 47%, females: 53%) than PAH (males: 25%, females: 75%) in our cohort (Prins et al., 2018). Furthermore, men with Group 3 PH have reduced RVFAC compared to women (26 ± 9 vs. 31 ± 11*%; p* = 0.028), and male sex is the strongest predictor of RV dysfunction in Group 3 PH even after adjusting for pulmonary vascular resistance and pulmonary arterial compliance (Prins et al., 2019). Finally, men exhibit a decline in RV contractility as pulmonary vascular resistance increases (*r* = −0.41, *p* = 0.04), but women do not (*r* = −0.04; *p* = 0.84) (Prins et al., 2019).

## Divergent RV Function Between Sexes in CTEPH (Group 4 PH)

Studies on sex dimorphisms in RV function in CTEPH or WHO Group 4 PH are limited. There are suggestions of superior RV function in females as there is enhanced long-term survival in females (>2 years after diagnosis) regardless of whether pulmonary endarterectomy is completed (annualized death rate of 6.0%, 95% CI: 4.5–7.6 vs. 7.7%, 95% CI: 5.9–9.5). However, it is uncertain whether this survival difference is due to differences in comorbidity burden (Barco et al., 2020). Studies of a Chinese CTEPH cohort (Chen et al., 2018) and the Spanish Registry of Pulmonary Arterial Hypertension, which includes CTEPH patients (Escribano-Subias et al., 2012) also show improved survival in females (male HR 1.38, 95% CI: 1.03–1.83*; p* = 0.03) (Escribano-Subias et al., 2012). In a Japanese cohort of CTEPH patients, females have significantly higher cardiac index (2.7 ± 0.6 vs. 2.4 ± 0.7 L·min^−1^·m^−2^; *p* = 0.01) and lower right atrial pressure (4 ± 4 vs. 7 ± 6 mmHg; *p* = 0.0002) compared to males despite no difference in pulmonary vascular resistance (Shigeta et al., 2008). While these results suggest enhanced RV function leads to better survival in females with CTEPH, further studies are needed to definitively evaluate this hypothesis.

## Sex Differences in ARVC/D

Multiple cohort studies from Europe (predominantly from the Netherlands) and the United States show that ARVC/D is 1.2–3 times more common in males (Corrado et al., 1997; Bauce et al., 2008; Cox et al., 2011; Bhonsale et al., 2015; Groeneweg et al., 2015). However, the male predominance is not observed in the North American ARVC Registry (89% of males vs. 84% of females in the ARVC registry diagnosed as affected, *p* = 0.424; 11% of males vs. 16% of females diagnosed as borderline ARVC, *p* = 0.424) (Choudhary et al., 2016), suggesting either a referral bias or there are differences between ethnic groups. Nevertheless, men with ARVC/D have an increased likelihood of severe ventricular arrhythmias (HR 2.76, 95% CI: 1.19–6.41) (Rigato et al., 2013), implantable cardioverter defibrillator therapies (HR 1.62, 95% CI: 1.20–2.19; *p* = 0.025) (Orgeron et al., 2017), and sudden cardiac death (relative risk 5.1, 95% CI: 3.0–8.5) (Hodgkinson et al., 2005; Bhonsale et al., 2015; Kimura et al., 2016; Lin et al., 2017; Bosman et al., 2018; Maupain et al., 2018; Dominguez et al., 2020). Males with ARVC/D more frequently have abnormal signal-averaged electrocardiograms, positive cardiac biopsies, inducible ventricular tachycardia/fibrillation, and have substrate properties, such as late potentials with longer duration of ventricular activities, larger epicardial unipolar low-voltage zones, and greater endocardial and epicardial areas with late potentials that increase risk of ventricular arrhythmias (Choudhary et al., 2016; Lin et al., 2017). Furthermore, men with ARVC/D have lower RV ejection fraction (50 ± 8 vs. 55 ± 6%; *p* < 0.0001) and higher RV end-diastolic volume (131 ± 30 vs. 119 ± 24% predicted; *p* = 0.004) as assessed by CMR (Sen-Chowdhry et al., 2007; Choudhary et al., 2016). The mechanisms of these sex dimorphisms are not well-defined and are hypothesized to be due to dissimilarities in competitive sports participation and/or effects of sex hormones.

## Estrogen: The Predominant Female Sex Hormone's Potential Protective Effects on the RV

### The Role of Estrogen on the RV in PAH

**Figure 2:**
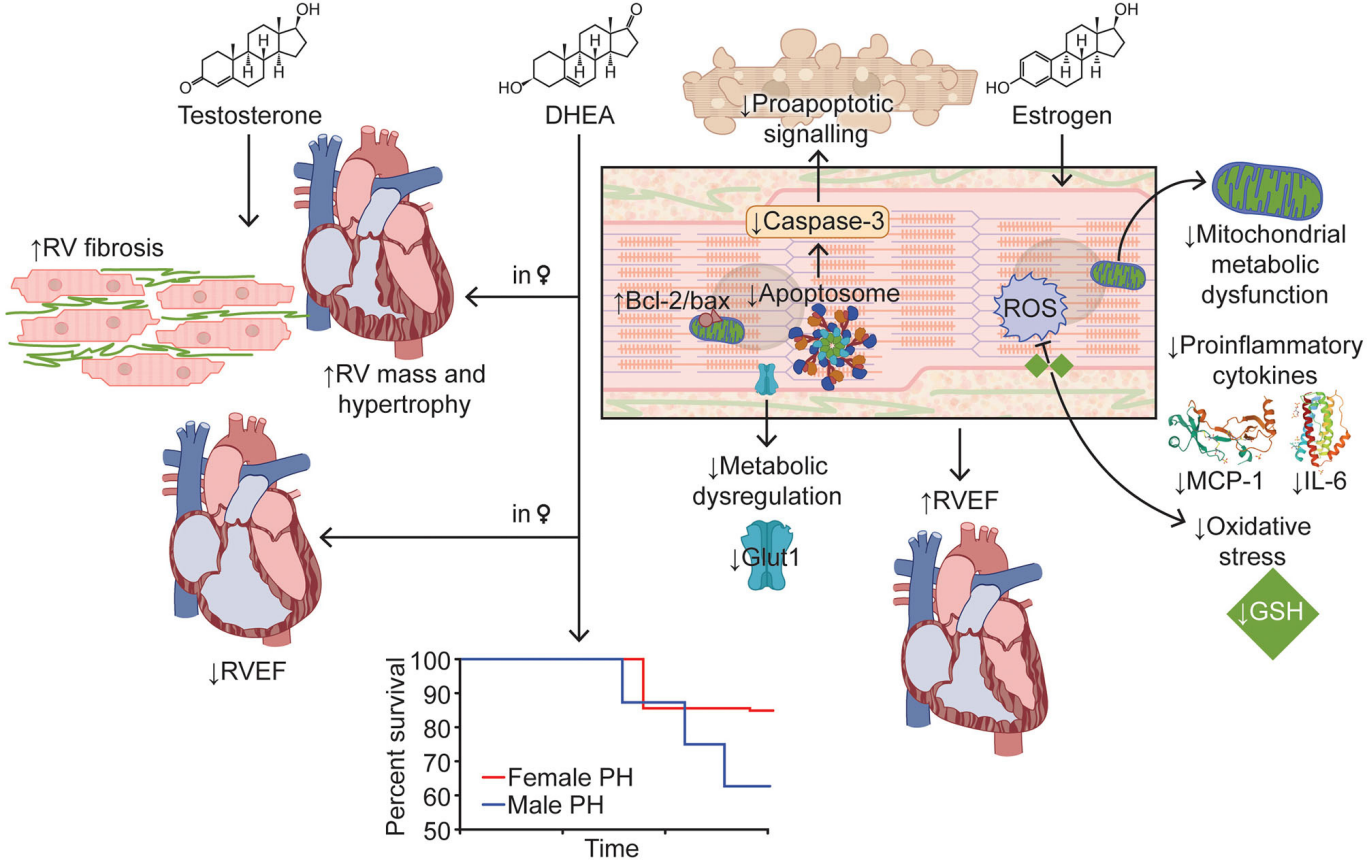
Effects of Sex Hormones on Right Ventricular Function in Pulmonary Hypertension. Estrogen improves RV function in PH via modulation of apoptosis, enhancement of mitochondrial function, and reduction in inflammation and oxidative stress. Testosterone increases RV mass, hypertrophy, and fibrosis, which results in decreased RV function. Higher DHEA levels in women are associated with lower RV ejection fraction and greater RV mass. Survival curve adapted from Rafikova et al. (2015). Images for MCP-1 and IL-6 adapted from Lubkowski et al. (1997) and Somers et al. (1997), respectively. Bcl-2/bax, B-cell lymphoma 2/bcl-2-associated X; DHEA, dehydroepiandrosterone; Glut1, glucose transporter 1; GSH, glutathione; IL-6, interleukin-6; MCP-1, monocyte chemoattractant protein-1; ROS, reactive oxygen species; RV, right ventricle/ventricular; RVEF, right ventricular ejection fraction.

In healthy women using hormone therapy, higher levels of the estrogen steroid hormone, estradiol corresponds with improved RV ejection fraction (β per 1 ln[nmol/L] 0.88, 95% CI: 0.32 to 1.43*; p* = 0.002) and lower RV end-systolic volume (RVESV) (β per 1 ln[nmol/L] −0.87, 95% CI: −1.67 to −0.08*; p* = 0.03) (Ventetuolo et al., 2011) (Figure 2). Thus, in the unloaded RV, estrogen has beneficial effects on RV form and function.

Our understanding of the effects of estrogen on RV function in PAH are guided by several prior compelling preclinical studies. Experimental models of PAH and RV dysfunction are summarized elsewhere (Lahm et al., 2018). Female Sugen (SU5416) hypoxia (SuHx) rats have better RV function compared to males with a lower percentage of females displaying RV outflow tract notching (75 vs. 100%) and females have a 1.9-fold higher cardiac index (*p* < 0.01) (Frump et al., 2015). The positive effects of estrogen are further supported by the fact that ovariectomy worsens RV function in female rats (Frump et al., 2015). In ovariectomized female and male SuHx animals, exogenous estrogen replacement enhances RV function, although this occurs in the setting of RV afterload mitigation (Liu et al., 2014; Frump et al., 2015; Lahm et al., 2016). Moreover, in male monocrotaline rats, estradiol treatment augments RV function and decreases RV hypertrophy and fibrosis (Wang et al., 2019).

Both estrogen receptor (ER)-α and ER-β agonists improve RV function, but the beneficial effects of estrogen on RV function are predominantly due to ER-α stimulation (Frump et al., 2015). This is supported by the finding that repletion of estradiol restores ER-α expression in ovariectomized SuHx rats (Frump et al., 2015). Additionally, there is a robust inverse correlation between ER-α expression and RV systolic pressure and RV hypertrophy and a positive association with cardiac output (Frump et al., 2015). In contrast, ER-β expression is not affected by sex, development of pulmonary hypertension, or estrogen withdrawal/repletion (Frump et al., 2015). Moreover, loss-of-function mutation in ER-α leads to RV-pulmonary arterial uncoupling, diastolic dysfunction, and more RV fibrosis in female, but not male, pulmonary artery banded rats, further demonstrating ER-α mediates superior RV adaptation in females (Cheng et al., 2020).

At the molecular level, estrogen supplementation has pleiotropic effects (Figure 2). First of all, estrogen augments RV mitochondrial function by mitigating the decrease in mitochondrial density and restoring expression of peroxisome proliferator-activated receptor gamma coactivator 1-α (Liu et al., 2017). Furthermore, estrogen repletion in ovariectomized SuHx rats attenuates alterations in RV glutathione activation, cytoplasmic glycolysis (decreasing glucose transporter 1 expression compared to ovariectomy), proapoptotic signaling (diminishing caspase-3 and increasing B-cell lymphoma 2 [bcl-2]/bcl-2-associated X [bax] signaling compared to ovariectomy), and expression of the proinflammatory cytokines, interleukin-6 and monocyte chemoattractant protein-1 (Frump et al., 2015). In summary, there are multiple lines of evidence that estrogen has a direct protective effect on RV function in preclinical PAH.

There are contradictory preclinical studies investigating the role of estrogen on PAH pathogenesis as some studies suggest exogenous estrogen and/or its metabolites aggravate pulmonary vascular remodeling (White et al., 2011; Dempsie et al., 2013) while others show protective effects (Umar et al., 2011; Philip et al., 2020). The importance and potential reasoning underlying these divergent findings are expertly reviewed elsewhere (Lahm et al., 2014). Certainly, the mechanistic effects of estrogen on the pulmonary vasculature in PAH need to be further elucidated to provide additional insight into how biological sex could impact RV afterload. Interestingly, higher levels of estradiol are linked to the development of PAH in men (OR: 54.9, 95% CI: 7.2–420.3*; p* < 0.001) (Ventetuolo et al., 2016), suggesting that estrogen may lead to increased susceptibility to PAH, but may confer better outcomes after the development of PAH due to improved RV function.

### The Role of Estrogen in ARVC/D

**Figure 3:**
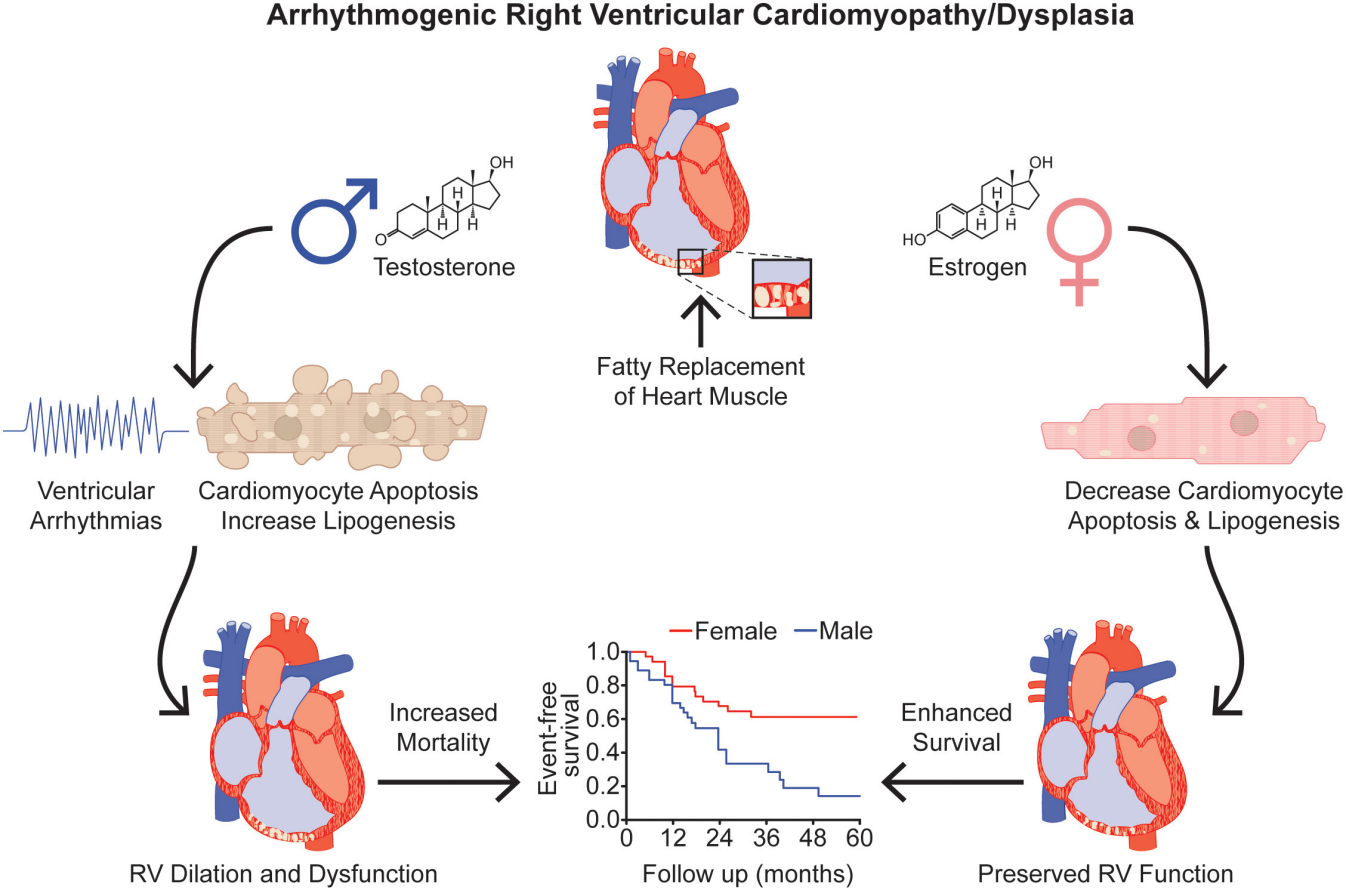
Effects of Sex Hormones in Arrhythmogenic Right Ventricular Cardiomyopathy/Dysplasia. Testosterone is associated with increased risk of severe ventricular arrhythmias, cardiomyocyte apoptosis, and lipid accumulation in ARVC/D. Conversely, estrogen decreases cardiomyoyte apoptosis and cardiomyocyte lipogenesis. Testosterone and estrogen's divergent effects on apoptosis and lipotoxicity provide a potential molecular understanding for the clinical observation that female ARVC/D patients have improved RV function and survival compared to males. Event-free survival curve adapted from Lin et al. (2017). RV, right ventricle/ventricular.

Decreased levels of the estrogen steroid hormone, estradiol are associated with major arrhythmic cardiovascular events in female ARVC/D patients (*p* = 0.03) (Akdis et al., 2017) (Figure 3). Moreover, when specifically evaluating female ARVC/D patients with desmosomal mutations, lower estradiol levels correspond with heightened risk of life-threatening arrhythmias (Akdis et al., 2017). Interestingly, in ARVC type V, the most aggressive heterozygous form of ARVC/D that is predominantly associated with a mutation (p.S358L) in transmembrane protein 43 (*TMEM43*) (a non-desmosomal gene), there is a signal that intense exercise increases ventricular arrhythmias only in females (*p* = 0.053) (Dominguez et al., 2020). This could partly be due to exercise lowering estrogen levels (Smith et al., 2013). Furthermore, the protective effect of estrogen may explain the later disease onset of ARVC/D in females (Rigato et al., 2013).

## Testosterone: The Main Male Sex Hormone's Possible Maladaptive Effects on the RV

### The Effects of Testosterone on the RV in PAH

In healthy males, higher testosterone levels are associated with greater RV mass (β per 1 ln[nmol/L] 0.44, 95% CI: 0.10–0.77; *p* = 0.01) and RVESV (β per 1 ln[nmol/L] 1.63, 95% CI: 0.55–2.71*; p* = 0.001) (Ventetuolo et al., 2011). In preclinical studies, male SuHx rats display exaggerated RV hypertrophy, more diffuse RV fibrosis, worse RV function, and consequently, higher mortality when compared to female SuHx rats (Rafikova et al., 2015) (Figure 2). In pulmonary artery banded male mice, castration decreases RV hypertrophy and improves survival from 40 to 100% at 10 months (*p* < 0.05) and trends toward mitigating markers of RV dysfunction in hemodynamic studies (decrease in end-diastolic pressure and time constant and increase in ejection fraction, cardiac output, and contractility index) (Hemnes et al., 2012). Repletion of testosterone in castrated mice reverses the positive RV effects, demonstrating the direct negative effects of testosterone on the RV (Hemnes et al., 2012). Castration also attenuates RV hypertrophy and fibrosis in male monocrotaline rats, but in the setting of diminished pulmonary vascular disease severity (Wen et al., 2019). Further studies are needed to understand the molecular mechanisms by which testosterone worsens RV function.

### The Effects of Testosterone in ARVC/D

Plasma testosterone levels are higher in ARVC/D patients compared to healthy controls (4.622 [0.467–7.433] vs. 1.988 [0.201–4.024] ng/mL; *p* < 0.0001; data expressed as median [IQR]) (Ren et al., 2020). Interestingly, males with ARVC/D have significantly elevated testosterone levels compared to healthy males (6.390 [4.438–8.768] vs. 3.617 [2.073–4.479] ng/ml; *p* < 0.0001; data presented as median [IQR]), but there are no differences in testosterone levels between ARVC/D and healthy females (Ren et al., 2020), suggesting that testosterone may modulate risk of adverse outcomes in ARVC/D. In fact, increased total and free testosterone levels are associated with greater risk of major arrhythmic cardiovascular events in males with ARVC/D (total testosterone 15.1 [0.5–19.5] vs. 7.9 [0.6–12.0] nmol/L; *p* = 0.01; data expressed as median [IQR] between ARVC/D patients with adverse vs. favorable outcomes) (Akdis et al., 2017; Ren et al., 2020) (Figure 3). Elevated testosterone levels are independently associated with heightened risk of malignant arrhythmic events in males with ARVC/D after adjusting for age, body mass index, RV and LV function, mutation status, antiarrhythmic drug use, Task Force diagnostic criteria, cardiovascular risk factors (e.g., smoking and alcohol use), and cardiovascular co-morbidities (Akdis et al., 2017; Ren et al., 2020). Of note, there are no differences in testosterone levels between sedentary males and males who participate in recreational or competitive sports, but there is a trend toward higher testosterone levels in males involved in competitive sports compared to sedentary males (*p* = 0.08) (Akdis et al., 2017).

In a seminal study using induced pluripotent stem cell-derived cardiomyocytes (iPSC-CMs) that harbor a mutation in the desmosomal protein, plakophilin-2 (c.2484C > T), physiological levels of testosterone increase cardiomyocyte apoptosis and lipid accumulation (Figure 3). Conversely, clinically relevant levels of estradiol decrease cardiomyocyte apoptosis and lipid accumulation (Akdis et al., 2017) (Figure 3). However, neither testosterone nor estradiol affects apoptosis or lipogenesis in normal iPSC-CMs (Akdis et al., 2017). These *in vitro* studies suggest that testosterone and estrogen directly modulate cardiomyocyte biology in ARVC/D patients. Furthermore, elevated testosterone levels heighten the risk of arrhythmogenesis by increasing adrenergic activity (Tsai et al., 2014). Additionally, testosterone modulates intracellular calcium handling (e.g., affecting the cardiac action potential which regulates calcium release from the sarcoplasmic reticulum, enhancing calcium release by the sarcoplasmic reticulum by augmenting the magnitude of individual calcium sparks, etc.) in isolated ventricular cardiomyocytes (Er et al., 2007; Ayaz and Howlett, 2015).

## Dehydroepiandrosterone (DHEA): A Potential Regulator of RV Function

### The Role of DHEA on the RV in PAH

DHEA, a precursor for androgens and estrogen, may also affect RV remodeling in PAH, but data regarding its direct effect on the RV are lacking. In human studies, higher DHEA levels in women are associated with lower RV ejection fraction (β per 1 ln[nmol/L] −0.54, 95% CI: −1.08–0.00; *p* = 0.05) and greater RV mass (β per 1 ln[nmol/L] 0.36; 95% CI: 0.10–0.62; *p* = 0.01) and RVESV (β per 1 ln[nmol/L] 1.43, 95% CI: 0.63–2.22*; p* < 0.001) (Ventetuolo et al., 2011) (Figure 2). Similar associations are observed in men, but they are not statistically significant (Ventetuolo et al., 2011). However, in PAH preclinical studies, DHEA has favorable effects on the pulmonary vasculature and RV. DHEA and DHEA sulfate (DHEA-S) decrease pulmonary arterial pressure, pulmonary vascular remodeling, and RV hypertrophy in chronic hypoxia and SuHx rats (Bonnet et al., 2003; Hampl et al., 2003; Oka et al., 2007; Alzoubi et al., 2013). Albeit, it is unclear whether the benefits on the RV are mostly due to decreased severity of PAH instead of a RV specific effect of DHEA.

There is one completed clinical trial and one ongoing clinical trial investigating the utility of DHEA in PH. In a pilot clinical trial of eight patients with Group 3 PH due to chronic obstructive pulmonary disease (NCT00581087), DHEA treatment for 3 months improved 6-min walk distance (390 [362–440] vs. 333 [257–378] m; *p* < 0.05; all data from this study shown as median [IQR] compared to baseline), mean pulmonary artery pressure (21 [20–22] vs. 26 [25–27] mmHg; *p* < 0.05), pulmonary vascular resistance (2.6 [2.5–3.8] vs. 4.2 [3.5–4.4] Wood units; *p* < 0.05), and carbon monoxide diffusing capacity of the lung (36.4 [14.6–39.6] vs. 27.4% [20.1–29.3] predicted; *p* < 0.05). Unfortunately, the effects of DHEA on the RV were not investigated (Dumas de La Roque et al., 2012). The influence of DHEA on RV function in PAH is currently being investigated in an ongoing phase 2 clinical trial (NCT03648385) (Table 1), and findings from this study will help clarify the direct effect of DHEA on the RV.

**Table 1:** Ongoing clinical trials targeting sex hormones in pulmonary arterial hypertension.

**Intervention**	**ClinicalTrials.gov identifier**	**Dose**	**Mechanism**	**Study start date**	**Estimated primary completion date**	**Estimated enrollment**	**Primary outcomes**	**Secondary outcomes**
Anastrozole	NCT03229499	1 mg PO daily for 1 year	Blocks conversion of androgens to estrogen	12/07/2017	9/2021	84	6MWD	- 6MWD
								- **RV function**
								- **NT-proBNP**
								- Biomarkers
								- SF36 and emPHasis-10 scores
								- Actigraphy-measured physical activity
								- Time-to-clinical worsening
								- Bone mineral density
								- Side effects
Tamoxifen	NCT03528902	20 mg PO TID for 24 weeks	Estrogen receptor binder (has both pro- and anti-estrogenic actions)	10/01/2018	6/30/2022	24	**TAPSE by echocardiogram**	- 6MWD
								- SF36 and emPHasis-10 scores
DHEA	NCT03648385	50 mg PO daily for 18 weeks	Endogenous precursor to androgens	1/09/2019	4/2023	24	**RV longitudinal strain by CMR**	- **RV ejection fraction by CMR**
								- **NT-proBNP**
								- Sex hormone levels
								- 6MWD
								- WHO functional class
								- SF36 and emPHasis-10 scores
								- Side effects

*The bold text describes direct and indirect measures of RV function. 6MWD, six minute walk distance; CMR, cardiac magnetic resonance imaging; DHEA, dehydroepiandrosterone; NT-proBNP, N-terminal pro b-type natriuretic peptide; PO, per os/by mouth; SF36, short form 36 health survey questionnaire; RV, right ventricle/ventricular; TAPSE, tricuspid annular plane systolic excursion; TID, ter in die/3 times a day; WHO, World Health Organization*.

### The Role of DHEA in ARVC/D

Studies on the role of DHEA and DHEA-S in ARVC/D are limited. However, in one study, lower levels of DHEA-S are associated with increased major arrhythmic cardiovascular events (3.1 [1.4–4] vs. 4.4 [1.9–7.2] mmol/L; *p* = 0.04; data expressed as median DHEA-S level [IQR] between ARVC/D patients with adverse vs. favorable outcomes) (Akdis et al., 2017). This association certainly needs to be validated in other cohorts and further studies are needed to determine the mechanisms by which lower DHEA or DHEA-S levels could lead to heightened arrhythmogenic risk in ARVC/D.

## Targeting Sex Hormones: Previous and Ongoing Clinical Trials in PAH

A trial completed in 2015 evaluated the effect of anastrozole, an aromatase inhibitor that blocks the conversion of androgens to estrogen, on RV function in PAH (NCT01545336) (Kawut et al., 2017). This was a study of 18 patients (12 patients given anastrozole 1 mg daily for 3 months and 6 patients given placebo) and there was no difference in change in tricuspid annular plane systolic excursion (TAPSE) (6.9 [0.0–27.8] vs. 10.0% [−5.8 to 33.3]; *p* = 0.98; data shown as median [IQR] change in TAPSE between the anastrozole and placebo groups) (Kawut et al., 2017). Another trial targeted estrogen signaling by investigating the utility of fulvestrant, a selective ER-α antagonist (given 500 mg intramuscularly on days 0, 14, 28, and 56; NCT02911844), in five patients. Fulvestrant did not change RV function as quantified by TAPSE (25 [18–27.5] vs. 19.5 [14.5–28.5] mm; *p* = 0.47; data presented as median [IQR] comparing follow-up vs. baseline measurements), RV index of myocardial performance (0.52 [0.37–0.82] vs. 0.29 [0.26–0.66]; *p* = 0.47), or stroke volume (62.2 [48–79.9] vs. 56 [36–73.5] mL; *p* = 0.07) after ~9 weeks follow-up (Kawut et al., 2019). Moreover, fulvestrant did not alter pulmonary vascular disease severity as assessed by RV systolic pressure (87 [40–89] vs. 86 [35–100] mmHg; *p* = 1.0) (Kawut et al., 2019). Thus far, these two trials have not demonstrated adverse RV effects of modulating estrogen signaling, but larger studies are needed to clarify the role of estrogen signaling in RV remodeling in PAH.

There are currently three ongoing clinical trials that are targeting sex hormone biology in PAH (Table 1). One trial is investigating how anastrozole (NCT03229499) affects 6-min walk distance. Plasma N-terminal pro b-type natriuretic peptide (NT-proBNP), an indirect assessment of RV overload and function, is a secondary outcome in this trial. Another trial is evaluating whether tamoxifen, an estrogen receptor binder that has both estrogenic and anti-estrogenic properties, alters TAPSE (NCT03528902). Finally, another ongoing trial is assessing whether DHEA affects RV longitudinal strain on CMR (NCT03648385). Hopefully, these trials will provide insights on whether estrogen and DHEA modulate RV function in human PAH.

## Conclusions

RV dysfunction is a strong predictor of poor outcomes in PH and ARVC/D, and thus there is a critical and unmet need for new RV-directed therapies to improve survival for these patients. There are clear differences in RV phenotypes between biological men and women with men having worse RV function in PH and ARVC/D and a higher risk of malignant ventricular arrhythmias in ARVC/D. Multiple preclinical studies suggest that these distinctions may be due to sex hormones as estrogen provides protective effects while testosterone has an adverse effect on the RV. Future studies that continue to investigate the direct effects of sex hormones on the RV and dissect the mechanisms leading to the sex differences in RV function in PH and ARVC/D will hopefully identify novel therapeutic targets for these devastating diseases. In addition to sex hormones, there are additional compounds that exhibit RV-enhancing activity in preclinical studies (Prisco et al., 2020). Because of the important biological discrepancies in RV function based on sex, we will need to consider how biological sex may modulate drug efficacy when translating these molecules to humans.

## Author Contributions

All authors completed a literature search, wrote sections of the manuscript, and designed the figures. All authors approved the submitted manuscript.

## Conflict of Interest

KP served as a consultant for Actelion and receives grant funding from United Therapeutics. The remaining authors declare that the research was conducted in the absence of any commercial or financial relationships that could be construed as a potential conflict of interest.
